# Co-application of bio-fertilizer and salicylic acid improves growth, photosynthetic pigments and stress tolerance in wheat under drought stress

**DOI:** 10.7717/peerj.9960

**Published:** 2020-10-27

**Authors:** Ammar Azmat, Humaira Yasmin, Muhammad Nadeem Hassan, Asia Nosheen, Rabia Naz, Muhammad Sajjad, Noshin Ilyas, Malik Nadeem Akhtar

**Affiliations:** 1 Department of Biosciences, COMSATS University Islamabad (CUI), Islamabad, Pakistan; 2 Department of Botany, PMAS-Arid Agriculture University, Rawalpindi, Pakistan

**Keywords:** PGPR, Salicylic acid, Drought, Wheat, Chlorophyll, Proteins, Antioxidants, Relative water content

## Abstract

Drought stress hampers the growth and productivity of wheat crop worldwide. Thus far, different strategies have been proposed to improve drought tolerance in wheat but the combined application of plant growth-promoting rhizobacteria formulated bio-fertilizer (BF) and salicylic acid (SA) has not been thoroughly explored yet. Therefore, a pot experiment was conducted to observe the effect of SA, BF, and their combination on wheat plants under optimal and drought stress conditions. Seeds priming was done with BF (10^7^ CFU mL^−1^). After 2 weeks of germination, SA (one mM) was applied as a foliar spray. Drought stress was applied by withholding water supply at three-leaf stage (30 d old plants) for the next 15 d until soil moisture dropped to 10%. Foliar application of SA increased the bacterial population of BF significantly compared to the sole application of BF under irrigated as well as drought stress conditions. Co-application of BF and foliar spray of SA induced drought tolerance in wheat plants by enhancing plant biomass, photosynthetic pigments, relative water content and osmolytes, and activities of the defense-related system. Plants treated with SA and BF together under drought stress had significantly increased leaf water status, Chl a, Chl b, and carotenoids synthesis by 238%, 125%, 167%, and 122%, respectively. Moreover, the co-application of SA and BF showed maximum SOD, POD, APX, and CAT activities by 165%, 85%, 156%, and 169% in the leaves while 153%, 86%, 116% and 200% in roots under drought stress. Similarly, the combined treatment exhibited a pronounced decrease in MDA content by 54% while increased production of proteins and proline by 145% and 149%, respectively. Our results showed that the co-application of SA and BF induced better drought tolerance as compared with the sole application of SA or BF. The results obtained herein suggest that combined application of BF and SA can be applied to the wheat crop to greatly improve drought tolerance in field conditions.

## Introduction

Drought is one of the most critical environmental stresses that reduce crop productivity (Khan et al., 2018). The agricultural production areas face a continuous decrease in irrigation water (Hafez, Ragab & Kobata, 2014). Crop productivity is reduced from 50% to 73% under limited water supply (Mehrdad, Sepideh & Hamed, 2017).

Bio-fertilizers are an important eco-friendly source of sustainable crop production. A bio-fertilizer comprising of one or more microorganisms improves the quantity and quality of growth and yield of the plants by increasing the availability of soil nutrients (Maria et al., 2018). A significant increase in plant resistance against abiotic stresses like drought, salinity, heat, and cold has been observed by plant growth-promoting rhizobacteria (PGPR) inoculation. The potential key processes of PGPR to alleviate drought stress includes 1-aminocyclopropane-1-carboxylic acid (ACC) deaminase activities to reduce the level of ethylene responsible for growth inhibition under drought stress conditions (Raghuwanshi & Prasad, 2018) phytohormones (indole acetic acid (IAA), gibberellic acid (GA), abscisic acid (ABA) and ethylene) production (Li et al., 2018), exopolysaccharides (EPS) production which increases the water holding capacity of the soil (Khan & Bano, 2016) and volatile organic compounds produced by rhizobacteria (Raza et al., 2015).

Salicylic acid (SA) is a phenol-based phytohormone and regulates vital plants’ physiological processes particularly water uptake and ion transport, transpiration, and photosynthesis (Klessig, Choi & Dempsey, 2018). SA crosstalk with other plant hormones and plant growth regulators to control different plant responses under normal as well as stress conditions (Raza et al., 2019). The interactions of SA with hormones such as brassinosteroids (Divi, Rahman & Krishna, 2010), GA (Roghayyeh et al., 2014), ABA (Jiang et al., 2010), IAA (Iglesias, Terrile & Casalongué, 2011), cytokinin (Jiang et al., 2013), and ethylene (Khan, Asgher & Khan, 2014) has been reported under optimal and stress conditions. Under stress conditions, the interactions of SA with various hormones can be ither synergistic or antagonistic. In barley roots, SA alleviated cadmium stress by reducing Cd-induced auxin-mediated reactive oxygen species (ROS) production. The authors suggested the role of SA in the IAA signaling pathway as the SA treatment reversed IAA-induced stress responses (Tamás et al., 2015). Similarly, an antagonistic interaction between SA and IAA was found in maize roots by Agtuca et al. (2014). The exogenous application of SA enhanced total root biomass while IAA improved lateral growth by offsetting primary root growth (Agtuca et al., 2014).

Exposure to different abiotic stresses, such as drought, enhances ethylene production and induces oxidative stress in plants (Khan et al., 2015). Enhanced ethylene production is due to the maximum expression of ethylene-responsive genes (Thao et al., 2015). SA treatment increased the ABA level in wheat seedlings under Cd stress due to a de novo ABA biosynthesis (Shakirova et al., 2016). Moreover, endogenous ABA regulated SA-mediated change in dehydrin protein concentrations under heavy metal stress, demonstrating a protective role of SA in wheat plants (Shakirova et al., 2016). Crosstalk between SA and jasmonates plays a critical role in plant growth regulation under abiotic conditions (Per et al., 2018). Normally, the signaling pathways of SA and jasmonic acid (JA) work in an antagonistic way (Khan & Khan, 2013). The antagonistic interaction between SA and JA cell signaling is mediated by the Mitogen-activated protein kinase (MAPK) genes (Petersen et al., 2000).

Studies have revealed that SA, an endogenous signal hormone is produced in a very low quantity and activates several physiological and biochemical processes in response to biotic and abiotic stressful conditions (Hafez, 2016). SA is also involved in the regulation of drought responses by enhancing the antioxidant capacity in plants (Saruhan, Saglam & Kadioglu, 2013).

It has been known that PGPR belonging to different genera and host plants are capable to alleviate abiotic stresses in plants by augmenting their defense system (Amna et al., 2020; Jochum et al., 2019; Yasmin et al., 2017). Similarly, the application of SA enhances tolerance to drought stress, thereby not only mitigating the damaging effects of drought stress but also enhancing its tolerance (Khan et al., 2018). However, the interactive effect of formulated bio-fertilizer and SA to increase drought tolerance in wheat plants by enhancing its defense responses specifically antioxidant enzyme activity is not well understood. It was hypothesized that the co-application of bio-fertilizer and SA can cause a delay in stress symptoms in wheat under water-stressed conditions. Therefore, this investigation was done to evaluate the effect of commercialized bio-fertilizer “Phytoguard” individually as well as in combination with SA on the growth, physiological and biochemical responses of wheat to observe the protective mechanism to water-deficient conditions.

## Materials and Methods

### Pot experiment

To evaluate the potential of bio-fertilizer and SA for ameliorating drought stress in wheat, a pot experiment was conducted in the greenhouse with rain exclusion shelter of COMSATS University, Islamabad (33.7294° N, 73.0931° E, Average temperature = 15 °C, humidity = 64%) during the growing season of wheat-October-December (2017). Seeds of wheat (*Triticum aestivum* L. cv. PAK 2013) were obtained from the National Agriculture Research Centre (NARC), Islamabad, Pakistan. Seeds were surface sterilized with 95% ethanol for 2 min then treated with 5% sodium hypochlorite for 1 min followed by successive washing with sterile distilled water. Commercialized bio-fertilizer (BF) “Phytoguard” (Wuhan Unioasis Biological Technology Co. LTD. (ISO 9001:2008)) was obtained from the local market. The “phytoguard” consists of a consortium of PGPR exhibiting phosphorous and potassium solubilization, nitrogen fixation, siderophore activity, and release of PGRs. The seeds were coated with bio-fertilizer (10^7^ CFU mL^−1^) mixed with 10% sugar solution used as a sticking agent. After 40 min, seeds were sown on ceramic trays containing sterilized sand. After 5 d of germination, the 15 seedlings were taken out and sown in pots.

The experiment was performed using a completely randomized design (CRD) with three replicates of each treatment. The plastic pots (6.2 cm (width) × 15.2 cm (length)) were filled with 3.7 Kg of sieved, sterilized, and air-dried soil and sand in a ratio of 3:1. The soil was sterilized by autoclaving it three times at an interval of 24 h between each cycle (Sinegani & Hosseinpur, 2010). The control group of pots was kept well-watered throughout the experiment. Pots with draining holes were watered with sterilized distilled water until the water seeped out of the pots from the bottom to reach the field capacity of 75–80%. After 7 d, thinning of the plants was done to keep 7 uniform seedlings per pot. After 2 weeks of germination, foliar application of SA (one mM) was done to the plants. This dose of SA was selected based on the previous experiment of Abd El-Mageed et al. (2016). Drought stress was applied by withholding water supply at three-leaf stage (30 d old plants) for the next 15 d until soil FC and moisture dropped to 40% and 10%, respectively. The watering of the drought-stressed group was done by measuring FC every day at the same time by weighing the pots and recompensing with the same volume of the water lost. However, FC of the non-stressed group was maintained at 75–80%. The description of all eight treatments used in this experiment is given in Table 1. Soil moisture content was observed by calculating the ratio between fresh and dry soil weight (Black, 1965). After 15 d of treatment, the plants were harvested. Roots and shoots were separated for biomass analysis that is, root and shoot length, fresh and dry weight, leaf area, and relative water contents. For different enzymatic assays, roots and shoots were collected separately per biological replicate and stored at 4 °C.

**Table 1:** Experimental work plan.

Symbol	Treatments
C	Irrigated control (Non-inoculated untreated seeds grown under irrigated conditions)
CD	Drought stress control (Non-inoculated untreated seeds grown under drought stress)
BF	Seeds primed with biofertilizer
BFD	Seeds primed with biofertilizer grown under drought stress
SA	SA control (foliar spray of SA)
SAD	Foliar spray of SA under drought stress
BF+SA	Seeds primed with BF+ foliar spray with SA
BF+SA+D	Seeds primed with BF+ foliar spray with SA under drought stress

### Colony-forming unit (CFU) g^−1^ rhizospheric soil

Rhizospheric soil was collected from each treatment for the estimation of viable cell count of applied inoculum. The colony-forming unit of rhizosphere soil was calculated using serial dilution and spread plate technique (Khan & Bano, 2016).

### Relative water content

After 15 d of drought stress induction, Relative water content (RWC) of wheat leaves was determined following Weatherley (1950). Fresh weight, dry weight, and fully turgid weight of fully turgid weight were recorded. Relative water content was calculated by the following formula:

$$\rm RWC\; \left( \% \right)\; = \; \left[ {\left( {\rm FW - \rm DW} ) / ( {\rm FTW - \rm DW} \right)} \right]\; \times \; 100$$

While, RWC = relative water content, DW = dry weight, FW = fresh weight, FTW = fully turgid weight.

### Total protein content

The total protein content of wheat leaves was determined as done by Lowry et al. (1951). Plant leaves (0.1 g) were ground and mixed with phosphate buffer. The solution was centrifuged at 3,000 rpm for 10 min. The resulting supernatant (0.1 mL) was mixed with distilled water to raise the volume up to 1 mL. This solution was mixed with the same volume of alkaline CuSO_4_ reagent and was shaken for 10 min. Finally, the Follin regent was added and the solution was incubated for 30 min at 28 ± 2 °C. Readings were measured at 650 nm. Bovine serum albumin (BSA) was taken as a reference for the calculation of total protein contents.

### Estimation of photosynthetic pigments

Photosynthetic pigments (chlorophyll (Chl) a, b, and carotenoids) of wheat leaves were estimated as defined by the Burnison (1980). Plant leaf (0.5 g) was mixed with 10 mL of dimethyl sulfoxide (DMSO) and the solution was heated at 65 °C in a water bath for 4 h. The supernatant was separated, and its absorbance was measured at 663 nm, 645 nm for Chl a, Chl b, and 480 nm for carotenoids, respectively.

### Estimation of proline content

Proline contents were quantified by using the protocol proposed by Bates, Waldren & Teare (1973). Wheat leaves (0.5 g) were ground in 80% ethanol. The resulting solution was heated at 80 °C for 1 h in a water bath. After centrifugation, 0.5 mL was extracted into a separate test tube. In supernatant, 0.5 mL d.H_2_O and 1 mL of 5% phenol were added and placed in an incubator for 1 h. After incubation, 2.5 mL sulfuric acid was mixed, and the readings were taken at 490 nm.

### Lipid peroxidation assay

To estimate lipid peroxidation of wheat leaves and root, malondialdehyde (MDA) content was measured by the thiobarbituric acid (TBA) assay as defined by Buege & Aust (1978). The 0.5 g of fresh leaves and roots of wheat plants were ground and mixed with the 100 mM phosphate buffer and the final volume was adjusted to 8 mL. The mixture was centrifuged at 20 min at 15,000 rpm at 4 °C and supernatant was used for further study. The reaction mixture was prepared with 5% trichloroacetic acid (TCA) and TBA. The 2.5 mL of the reaction mixture were added to 1.5 mL of enzyme extract. The resulting solution was heated at 100 °C for 30 min in a water bath. The solution was allowed to cool down and was centrifuged at 4,800 rpm for 10 min. The supernatant was used for measuring the absorbance at 532 nm. The reaction mixture of TBA and 5% TCA was taken as control.

### Antioxidants enzymes activities

#### Enzyme extracts

For enzyme extract preparation, 0.5 g leaves and roots were ground in 3 mL phosphate buffer (pH. 7.8). The sample was homogenized on ice and the solution was raised to 5 mL before centrifugation at 13,000 rpm for 20 min at 4 °C. The supernatant was covered with aluminum foil to avoid exposure to light and was preserved at 4 °C for different enzymatic assays.

#### Superoxide dismutase

To determine Superoxide dismutase (SOD) activity, the procedure established by Beyer & Fridovich (1987) (EC 1.15.1.1) was used. SOD enzyme was used to measure the inhibition in the photoreduction of nitro blue tetrazolium (NBT). Reaction mixture was prepared with a pH 7.6 sodium phosphate buffer (50 mM), EDTA (0.1 mM), sodium carbonate (50 mM), L-methionine (12 mM), NBT (50 μM), riboflavin (10 μM) and crude extract (100 μL) and the final volume was 3.0 mL.

For comparison, a set of reactions with all components except the crude extract was taken as control. Initially, the reaction tubes were exposed to white light for 15 min for starting the reactions. Reactions were stopped by switching off the lights and readings were measured at 560 nm (Oktay et al., 1995).

#### Peroxidase activity

Peroxidase Activity (POD) (EC# 1.11.1.x) activity of wheat leaves was measured by the procedure of Gorin & Heidema (1976). In this method, 4-methyl catechol was used as a substrate that causes oxidation when mixed with H_2_O_2_. The reaction mixture contained 100 mM sodium phosphate (Na_3_PO_4_) buffer with pH 7.0, 5 mM 4-methyl catechol, 5 mM H_2_O_2,_ and 500 μL of crude extract resulting in a solution with a total volume of 3.0 mL. Readings were recorded at 420 nm.

#### Ascorbate peroxidase

Ascorbate peroxidase (APX) (EC# 1.11.1.11) activity was estimated by the method of Asada & Takahashi (1987) through monitoring the rate of ascorbate oxidation at a wavelength of 290 nm. The reaction mixture contained a pH 7 phosphate buffer (50 mM), H_2_O_2_ (0.1 mM), ascorbic acid (0.5 mM), an enzyme extract (100 µL) of the sample.

#### Catalase activity

Catalase Activity (CAT) (EC# 1.11.1.6) activity was done by the technique of Kumar et al. (2010). The reaction mixture containing 0.1 mL of 300 mM H_2_O_2,_ 2.8 mL of 50 mM phosphate buffer along with 0.1 mL of enzyme solution was prepared. The absorbance of the assay mixture was measured at 240 nm after intervals of 30 s.

### Statistical analysis

Statistical analysis of data was done by applying one-way analysis of variance (ANOVA) suitable to CRD, correlation coefficient, and comparison between mean values was made using Statistix 8.1 (Analytical Software, Cary, NC, USA). A significant difference among treatments was carried out by using the least significant difference (LSD) at a significance level of *P* ≤ 0.05 as defined by Steel & Torrie (1980). Web tool clustvis (https://biit.cs.ut.ee/clustvis/) was used for preparing the heatmap of the correlation coefficient.

## Results

### Colony-forming unit of rhizospheric soil (CFU g^−1^)

Drought stress significantly reduced the rhizospheric CFU of wheat plants inoculated with BF as compared to the non- inoculated control plants (Table 2). Foliar application of SA increased the CFU significantly than the sole application of BF under irrigated as well as drought-stressed conditions (Table 3).

**Table 2:** Effect of biofertilizer (Phytoguard) and salicylic acid (SA) on the fresh and dry weight of shoot and roots of wheat (*Triticum aestivum* L.) plants in control and drought-stressed conditions.

Treatments	Shoot fresh weight (g)	Shoot dry weight (g)	Root fresh weight (g)	Root dry weight (g)
C	0.13 ± 0.019^E^	0.036 ± 0.013^F^	0.078 ± 0.0006^F^	0.033 ± 0.0012^E^
CD	0.13 ± 0.002^E^	0.035 ± 0.002^F^	0.22 ± 0.001^D^	0.04 ± 0.0003^E^
BF	0.35 ± 0.003^B^	0.18 ± 0.003^C^	0.26 ± 0.020^D^	0.078 ± 0.004^CD^
BF+D	0.28 ± 0.006^C^	0.12 ± 0.003^DE^	0.53 ± 0.0016^B^	0.28 ± 0.003^B^
SA	0.31 ± 0.006^C^	0.14 ± 0.001^D^	0.16 ± 0.015^E^	0.07 ± 0.0014^D^
SA+D	0.22 ± 0.005^D^	0.11 ± 0.005^E^	0.47 ± 0.006^C^	0.21 ± 0.0008^B^
BF+SA	0.41 ± 0.007^A^	0.25 ± 0.011^A^	0.44 ± 0.0016^C^	0.087 ± 0.003^C^
BF+SA+D	0.34 ± 0.010^B^	0.20 ± 0.0027^B^	0.613 ± 0.0076^A^	0.32 ± 0.006^A^

**Note:**

Values are the means ± SE (*n* = 3). A different alphabet after each data within the same column exhibit the significant difference at (*P* = 0.05).

**Table 3:** Effect of biofertilizer (Phytoguard) and salicylic acid (SA) on the length of shoot and roots, leaf area and CFU g^−1^ soil of wheat (*Triticum aestivum* L.) plants in control and drought stress.

Treatments	Shoot length (cm)	Root length (cm)	Leaf area (g)	CFU g^−1^ soil
C	24.9 ± 0.46^F^	10.3 ± 0.11^F^	12.7 ± 0.37^D^	0
CD	18.3 ± 0.70^G^	13.06 ± 0.44^E^	10.5 ± 0.68^E^	0
BF	30.5 ± 0.32^B^	17.7 ± 0.06^BC^	17.9 ± 0.54^B^	1.49 × 10^6^
BF+D	28.2 ± 0.14^D^	17.16 ± 0.08^CD^	14.7 ± 0.11^C^	1.38 × 10^6^
SA	29.4 ± 0.12^BC^	16.6 ± 0.06^D^	18.3 ± 0.27^B^	0
SA+D	26.4 ± 0.21^E^	17.2 ± 0.31^C^	12.6 ± 0.15^D^	0
BF+SA	34.8 ± 0.45^A^	19.6 ± 0.06^A^	19.6 ± 0.22^A^	1.54 × 10^6^0
BF+SA+D	29 ± 0.12^CD^	18.1 ± 0.1^B^	18.4 ± 0.23^B^	1.48 × 10^6^

**Note:**

Values are the means ± SE (*n* = 3). A different alphabet after each data within the same column exhibit the significant difference at (*p* = 0.05).

### Influence of salicylic acid and bio-fertilizer application on growth of wheat (*Triticum aestivum* L.)

**Figure 1:**
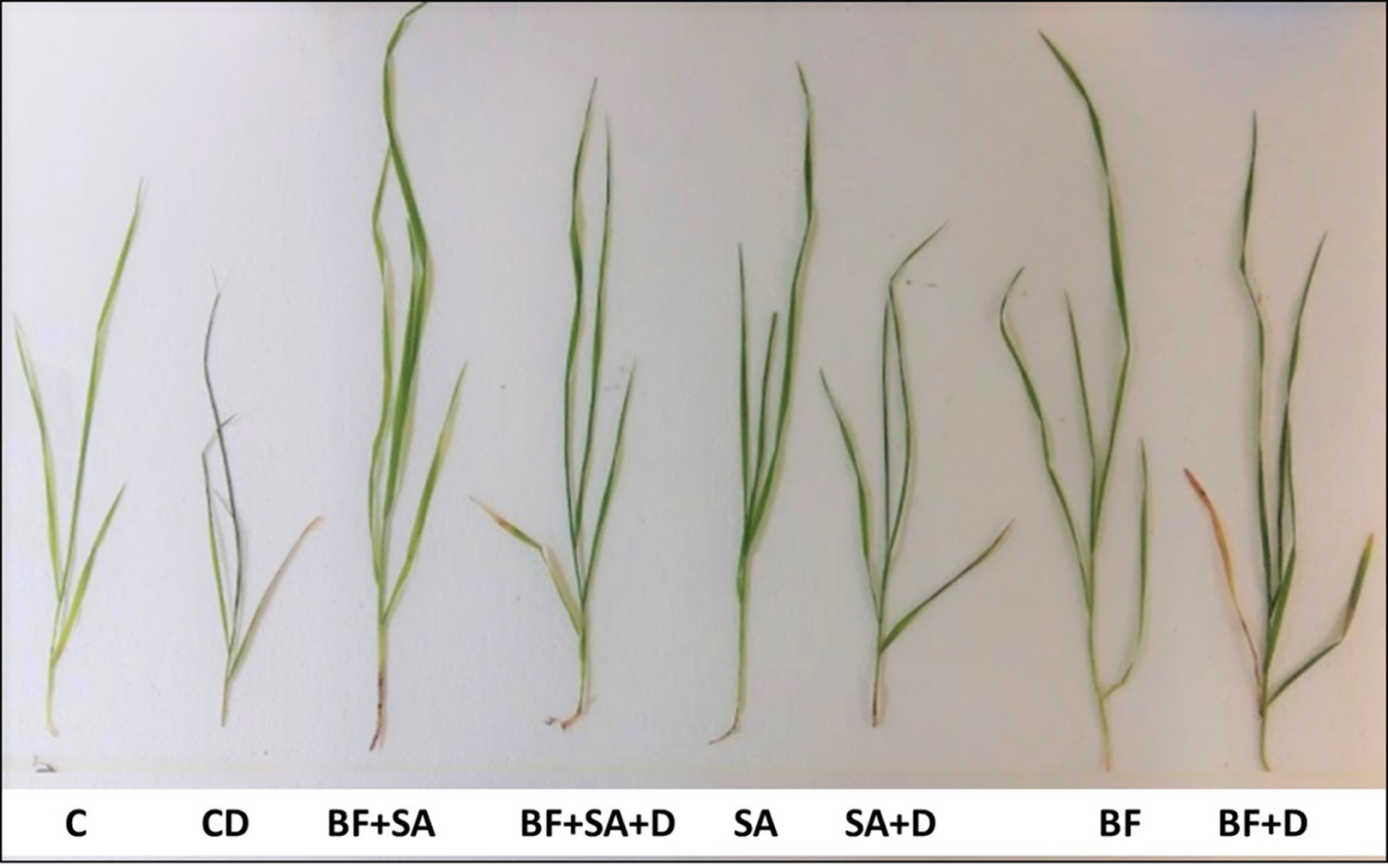
Effect of foliar spray of salicylic acid (SA) and bio-fertilizer (BF) inoculation on growth of wheat *(Triticum aestivum* L.) plant under drought stressed condition. Whereas; C, Control; CD, Drought stress; BF+SA, Combined application of biofertilizer and salicylic acid; BF+SA+D, Combined application of biofertilizer and salicylic acid under drought stress; SA, salicylic acid; SA+D, salicylic acid under drought stress; BF, Biofertilizer, BF+D, Biofertilizer under drought stress.

Drought mitigating potential of BF (Phytoguard) and foliar spray of SA was observed for the wheat (*Triticum aestivum* L.) seedlings grown under drought-stressed conditions. Exposure of wheat seedlings to drought stress negatively affected the germination and growth of wheat seedlings as shown in (Fig. 1). The seedlings showed less biomass, decreased vigor, wilting of leaves, and premature senescence. After 15 d exposure to drought stress, the plants showed visible effects of drought stress as compared to the control.

### Plant biomass

Application of SA and BF, sole and in combination, resulted in more biomass and leaf area in wheat plants under irrigated and drought-stressed conditions as compared to their non-inoculated control (Tables 2 and 3). Under drought stress conditions, the combined application of SA and BF showed maximum increase in shoot and root fresh weight by 171% and 175% and dry weight by 495% and 677% respectively, as compared with non-inoculated control (Table 2)

Results in Table 3 displayed that all treated plants had a significant increase in shoot and root length under drought stress conditions. However, the combined application of SA and BF caused a maximum of 58% and 38% increase in shoot and root length respectively, as compared to the non-inoculated control in drought-stressed plants. The combined treatment of SA and BF increased the leaf area by 54% as compared to the non-inoculated control under non- stressed conditions (Table 3).

### Relative water content

All the treated plants showed a marked rise in RWC of leaves than the non-treated control in both irrigated range from 11.14% to 18.2% and stressed group range from 154.75% to 238%. However, drought severely affected the water status of wheat seedlings depicted by reduced RWC by 78% than the irrigated control. Plants treated with SA and BF together under drought stress had significantly increased leaf water status by 238% of wheat plants as compared to the non-inoculated control under non-stressed conditions (Table 3).

### Protein content

**Figure 2:**
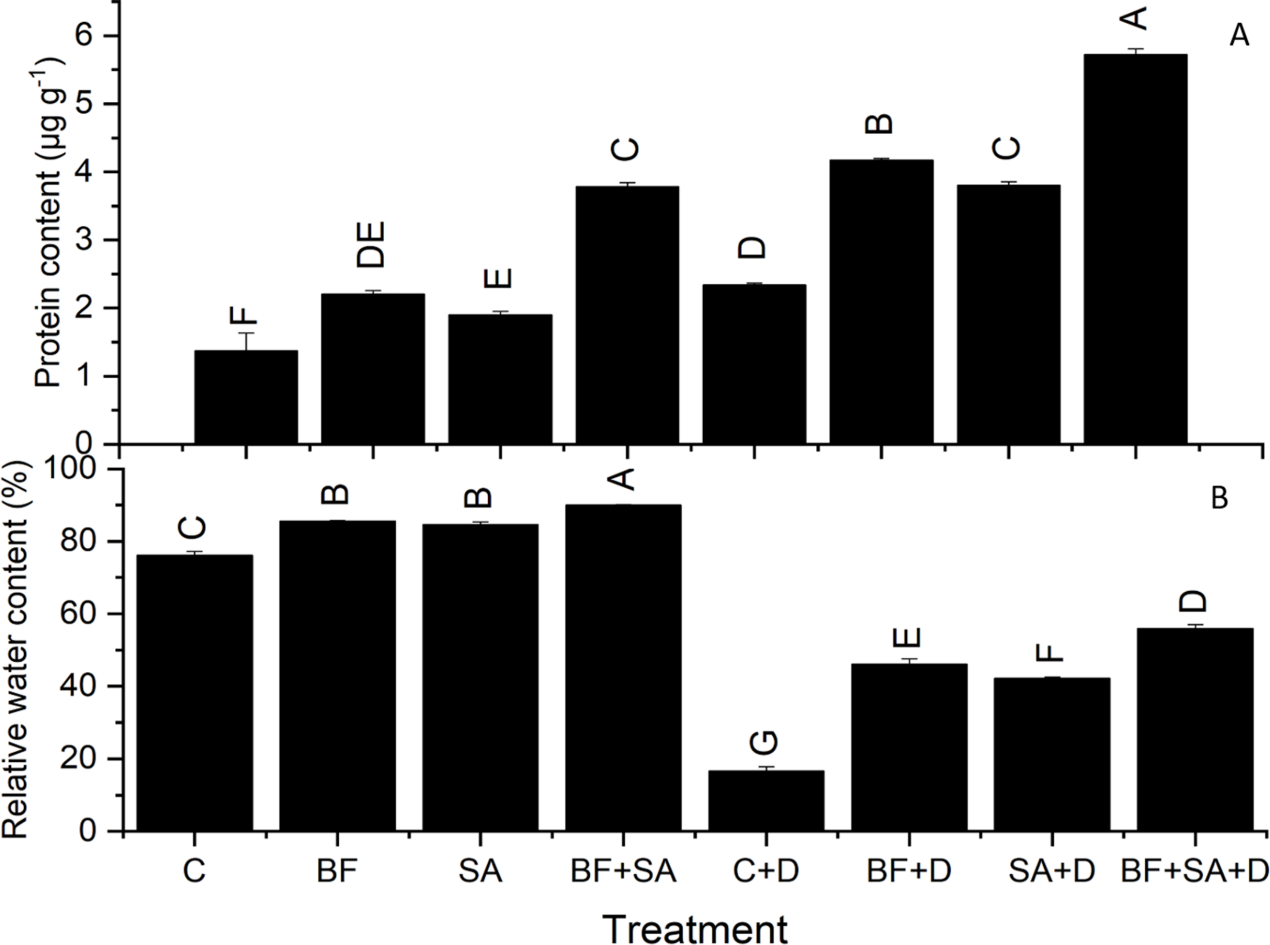
Effect of biofetilizer (Phytoguard) and salicylic acid (SA) on (A) the relative water content and (B) total protein contents of wheat (*Triticum aestivum* L.) plants in control and drought stressed conditions. All the values are the mean of three replicates ± standard error of means. Different letters indicate statistically significant difference between treatments (*P* ≤ 0.05). Details of treatments as given in Fig. 1.

All treatments resulted in a significant increase in protein synthesis of wheat seedlings range from 2.2–3.7 µg g^−1^ under irrigated conditions 4.2–5.72 µg g^−1^ under drought stress than non-inoculated plants. In the drought-stressed plants, the higher protein synthesis was recorded (2.3 µg g^−1^) as compared to the irrigated control plants (1.36 µg g^−1^). Under the non-stressed conditions, the maximum production of protein was observed by the combined application of SA and BF (3.7 µg g^−1^). However, under the water-stressed conditions, SA and BF together alleviated the effect of drought by increasing the protein content (5.72 µg g^−1^) shoots as compared to the non-inoculated drought-stressed plants (2.3 µg g^−1^) (Fig. 2).

### Photosynthetic pigments

The Chl a, Chl b, and carotenoid contents of the wheat plant were significantly decreased by 31%, 48%, and 42% respectively, under induced drought stress than the non-inoculated control plants.

All the treated seedlings showed increased Chl a, Chl b, and carotenoid contents range from 48% to 70%, 42% to 89%, and 6% to 43% than the non-treated irrigated control. Similarly, under drought stress, SA and BF exhibited a pronounced increase in the contents of Chl a, Chl b, and carotenoid ranges from 88% to 125%, 74% to 168%, and 85% to 125% in both sole and combined treatments than the drought stress control.

**Figure 3:**
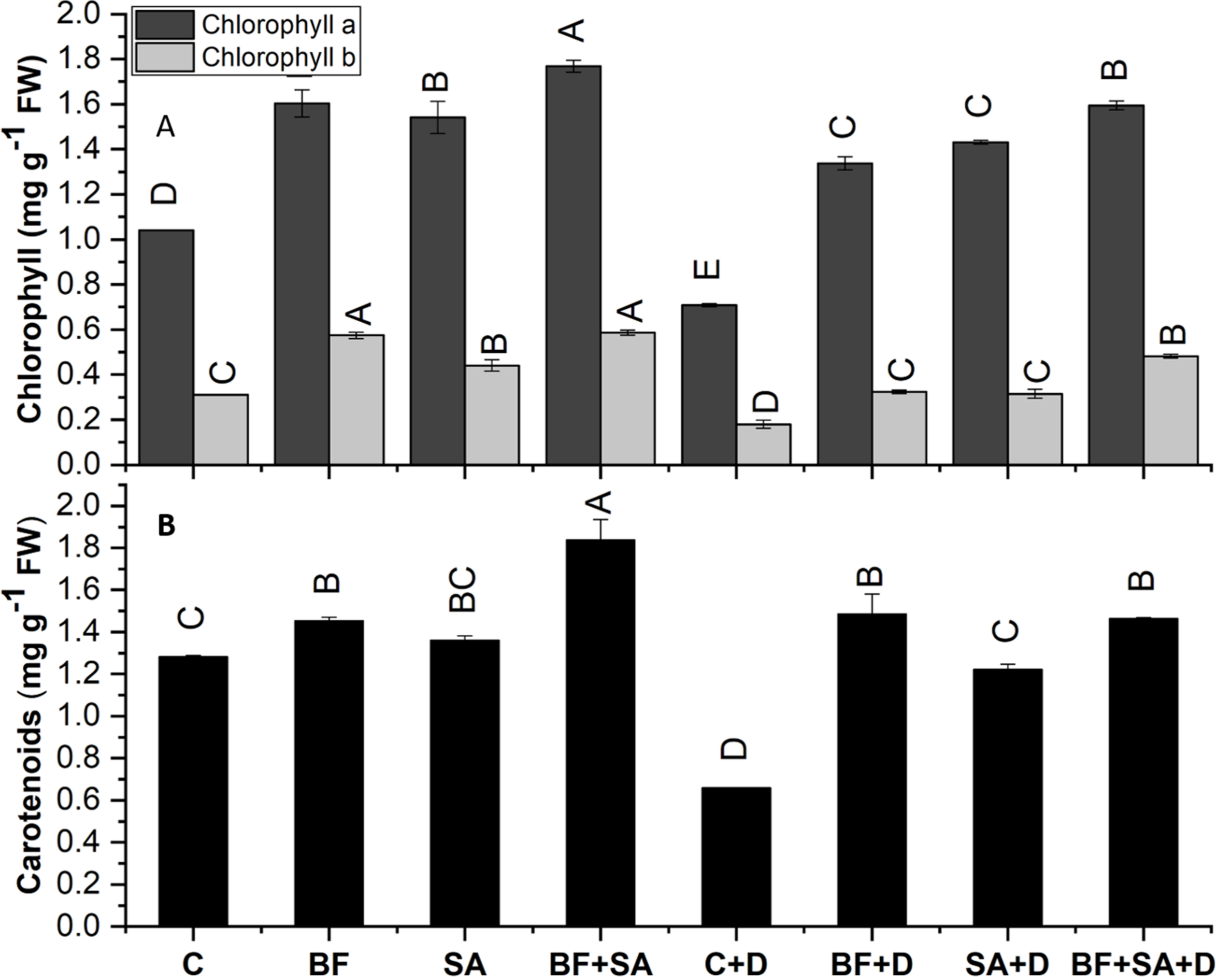
Effect of biofetilizer (Phytoguard) and salicylic acid (SA) on (A) chlorophyll and (B) carotenoids content of wheat (*Triticum aestivum* L.) plants in control and drought stressed conditions All the values are the mean of three replicates ± standard error of means. Different letters indicate statistically significant difference between treatments (*P* ≤ 0.05). Details of treatments as given in Fig. 1.

The combined application of SA and BF showed an increase in Chl a, Chl b, and carotenoid synthesis by 125%, 167%, and 122% respectively, as compared to the non-inoculated under drought (Fig. 3).

### Malondialdehyde

All treatments caused a significant reduction in MDA contents in shoots (32–54.2%) as well as in roots (13.5–52.4%) as compared to the non-inoculated control plants under drought stress. In drought-stressed plants, the MDA level was increased by 211% in shoots and 91% in roots as compared to the control plants.

**Figure 4:**
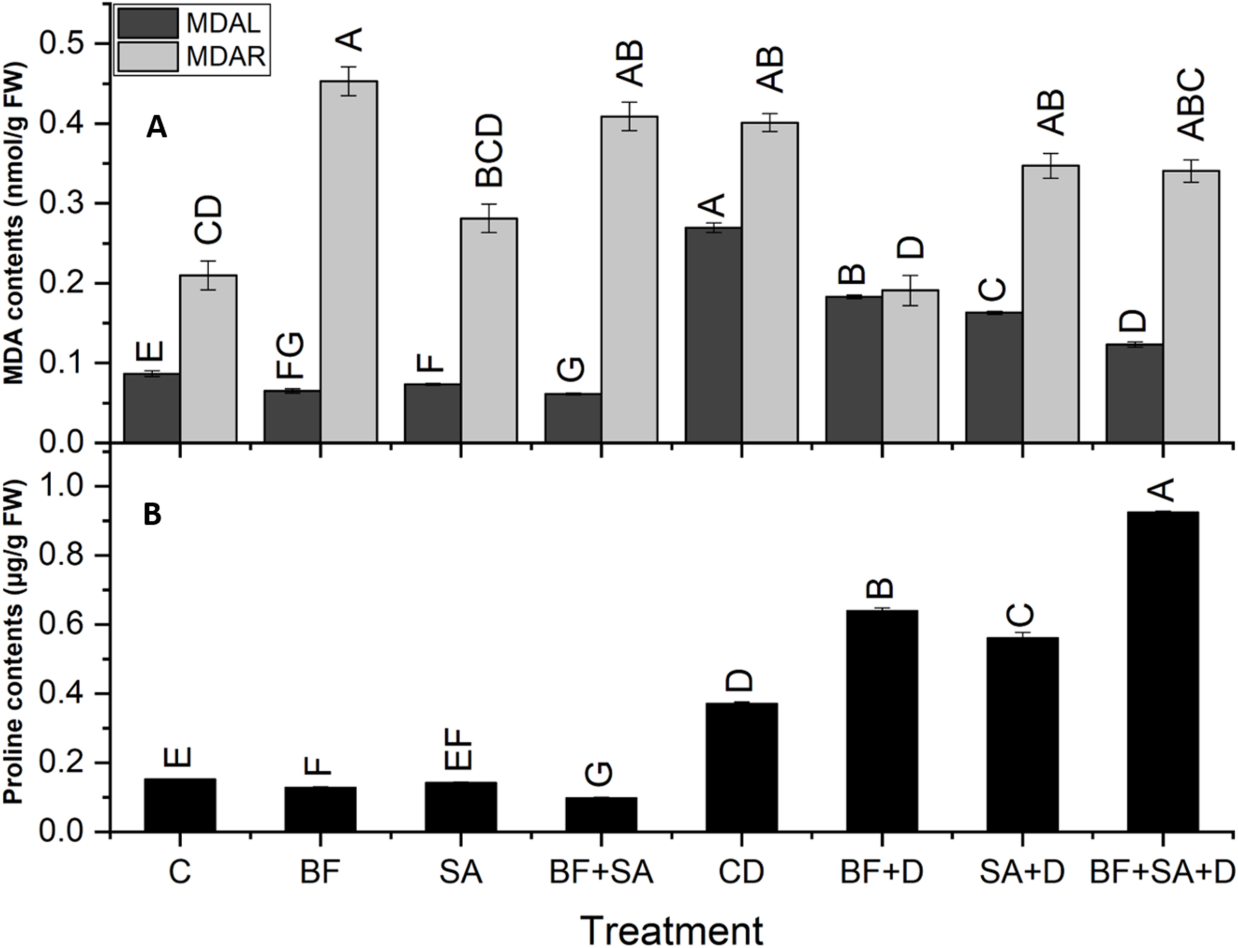
Effect of biofetilizer (Phytoguard) and salicylic acid (SA) on (A) MDA content and (B) proline contents of wheat (*Triticum aestivum* L.) plants in control and drought stressed conditions. All the values are the mean of three replicates ± standard error of means. Different letters indicate statistically significant difference between treatments (*P* ≤ 0.05). Details of treatments as given in Fig. 1.

The combined application of SA and BF significantly decreased the level of MDA contents in shoots by 54% as compared to non-inoculated drought-stressed plants. However, the sole application of BF showed a maximum significant decrease in roots by 52% as compared to the non-inoculated plants under drought stress (Fig. 4).

### Estimation of proline

The level of shoot proline significantly increased in the BF sole and combined treatment of SA and BF as compared to the non-inoculated control under water stress conditions. Under drought stress, plants showed a 145% increase in proline contents as compared to the control plants. Under normal conditions, a pronounced decrease was revealed by the combined treatment by 35% in proline synthesis as compared to the non-inoculated control plants. However, under drought stress treatments, the combined treatment with BF and SA resulted in a 149% increase in the proline synthesis as compared with the non-inoculated drought-stressed plants (Fig. 4).

### ROS scavenging enzymes

**Figure 5:**
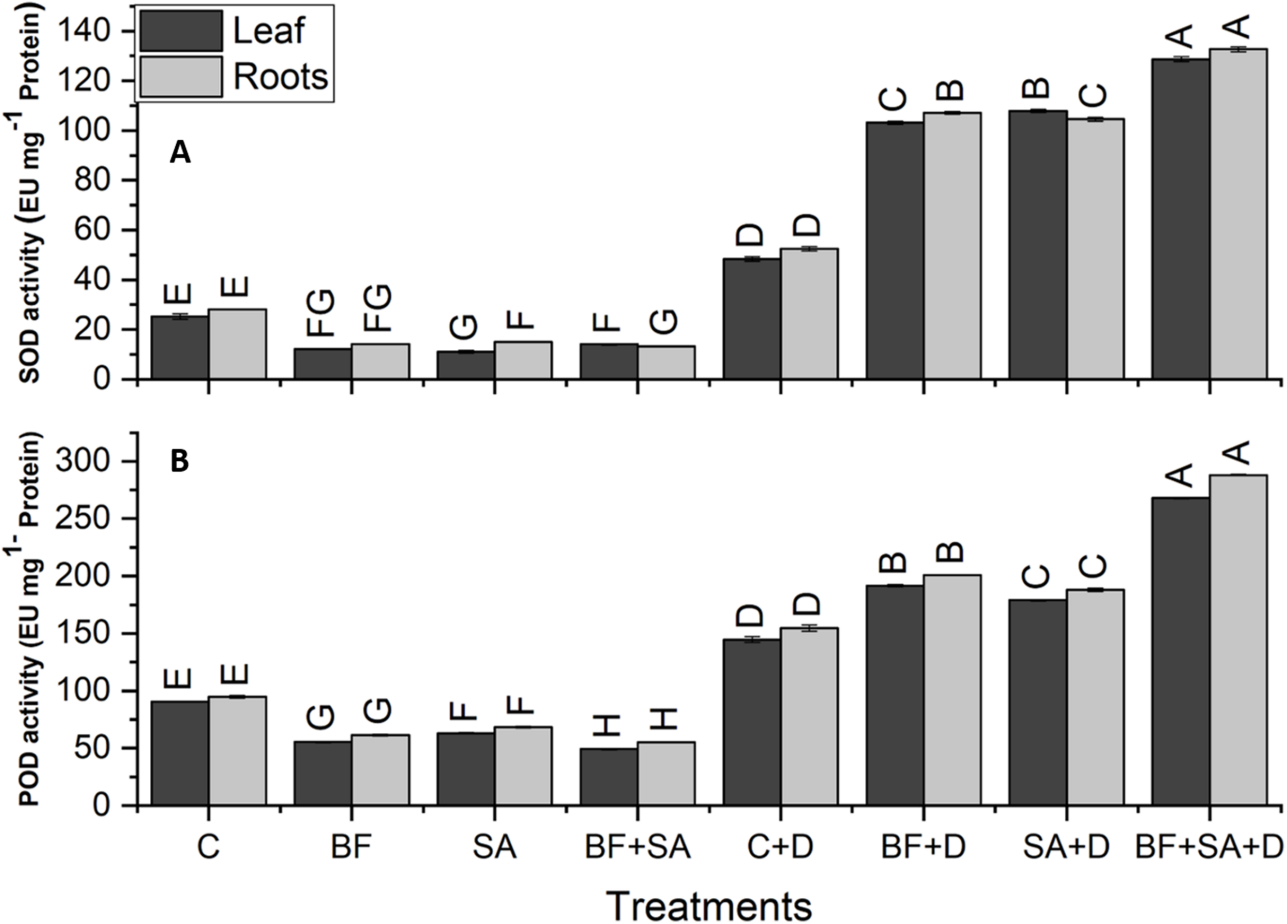
Effect of biofetilizer (Phytoguard) and salicylic acid (SA) on (A) SOD activity and (B) POD activity of wheat (*Triticum aestivum* L.) plants in control and drought stressed conditions. All the values are the mean of three replicates ± standard error of means. Different letters indicate statistically significant difference between treatments (*P* ≤ 0.05). Details of treatments as given in Fig. 1.

**Figure 6:**
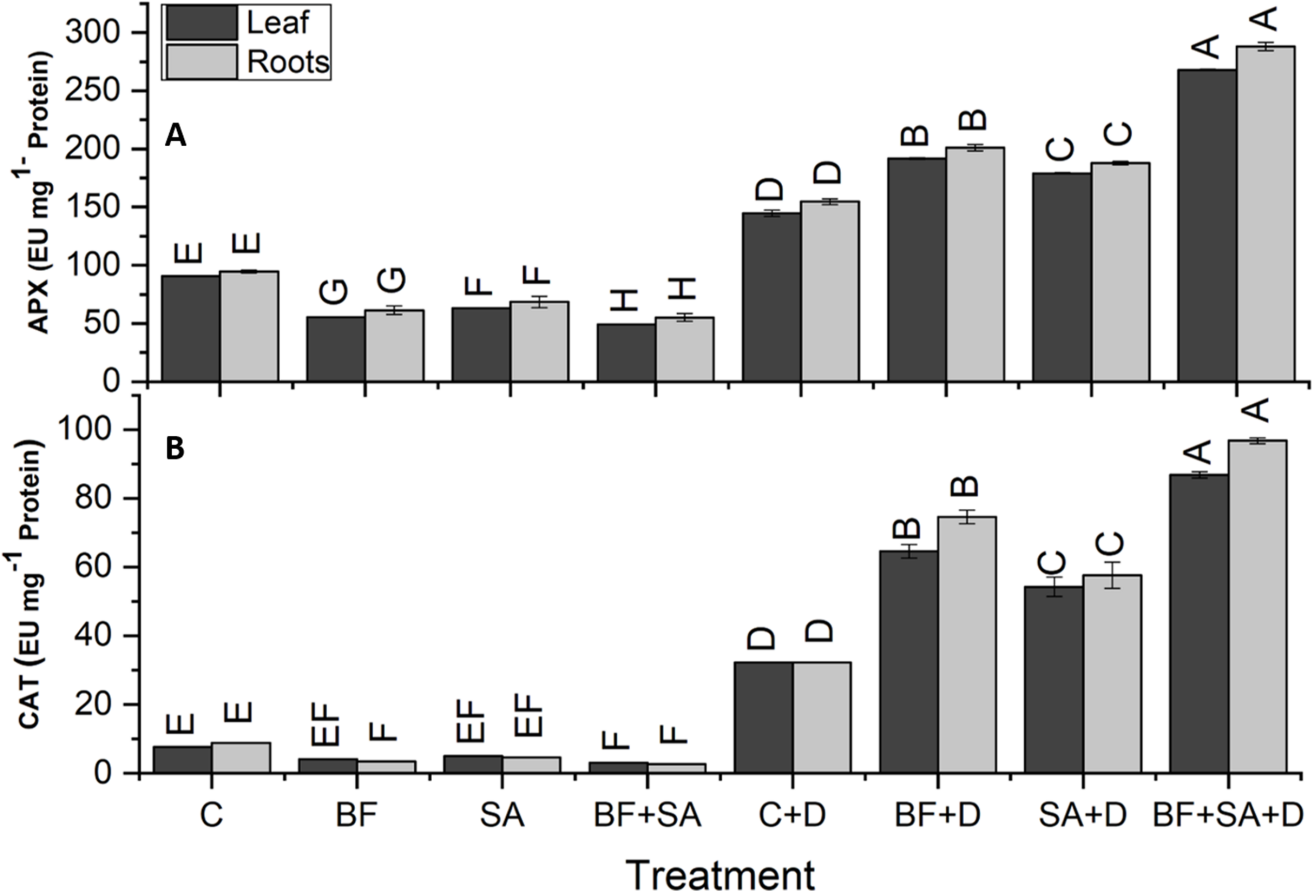
Effect of biofetilizer (Phytoguard) and salicylic acid (SA) on enzyme activity of (A) APX and (B) CAT of wheat (*Triticum aestivum* L.) plants in control and drought stressed conditions. All the values are the mean of three replica. Different letters indicate statistically significant difference between treatments (*P* ≤ 0.05). Details of treatments as given in Fig. 1.

The drought-stressed plants showed 91%, 59%, 94%, and 321% increase in SOD, POD, APX, and CAT activities of leaves respectively, as compared with the irrigated control. However, in roots, the drought-stressed plants showed 86%, 63%, 103%, and 262% increase in SOD, POD, APX, and CAT activities respectively, as compared to the irrigated control (Figs. 5 and 6).

Application of BF and SA significantly decreased antioxidants enzymes activities in wheat plants when treated sole or in combination as compared to the non-treated control in the roots and shoots of well-watered seedlings. However, under drought stress conditions, all treatment of SA and BF was effective to alleviate the effects of drought stress by increasing activities of SOD, POD, APX, and CAT. However, combined application SA and BF were more effective than the sole treatments both in roots and shoots. Combined treatment of SA and BF showed maximum SOD, POD, APX, and CAT activities by 165%, 85%, 156%, and 169% in the leaves as compared to the non-inoculated control. In roots, significantly higher activities of SOD, POD, APX, and CAT were recorded by 153%, 86%, 116%, and 200%, which was induced by combined treatment of SA and BF as compared with the non-inoculated control under (Figs. 5 and 6).

### Heatmap responses of Pearson’s Correlation Coefficient (r)

**Figure 7:**
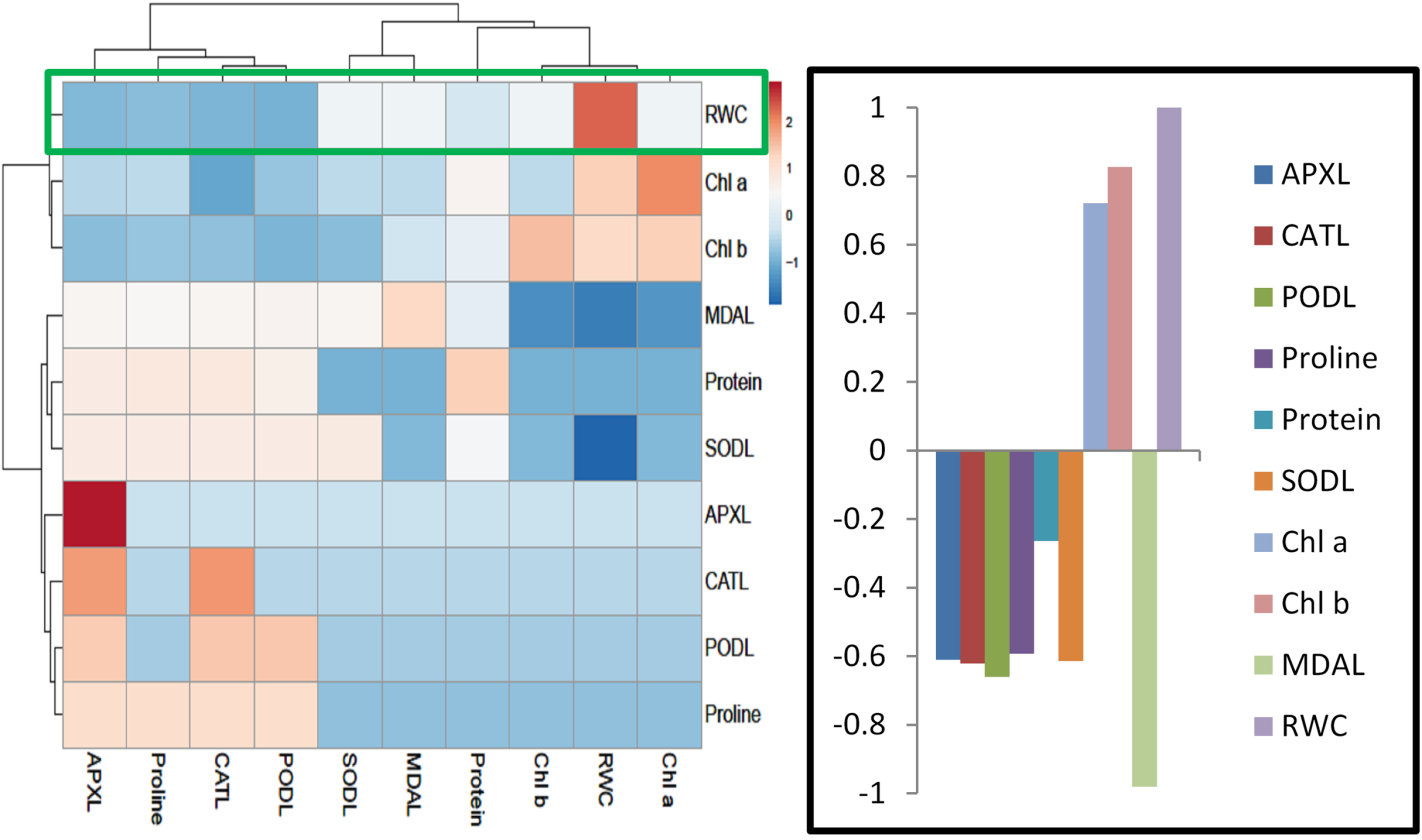
Heatmap responses of Pearson’s Correlation Coefficient (r) for the antioxidant enzymes, stress determinants and relative water content of wheat leaves treated with sole and combined application of SA and BF under drought stressed condition. APXL, leaves ascorbate peroxidase; CATL, leaves catalase; PODL, leaves peroxidase; SODL, leaves superoxide dismutase; chla, chlorophyl “a”; chlb, chlorophyl “b”; MDA leaves, leaves Melondialdehyde; RWC, relative water content.

**Figure 8:**
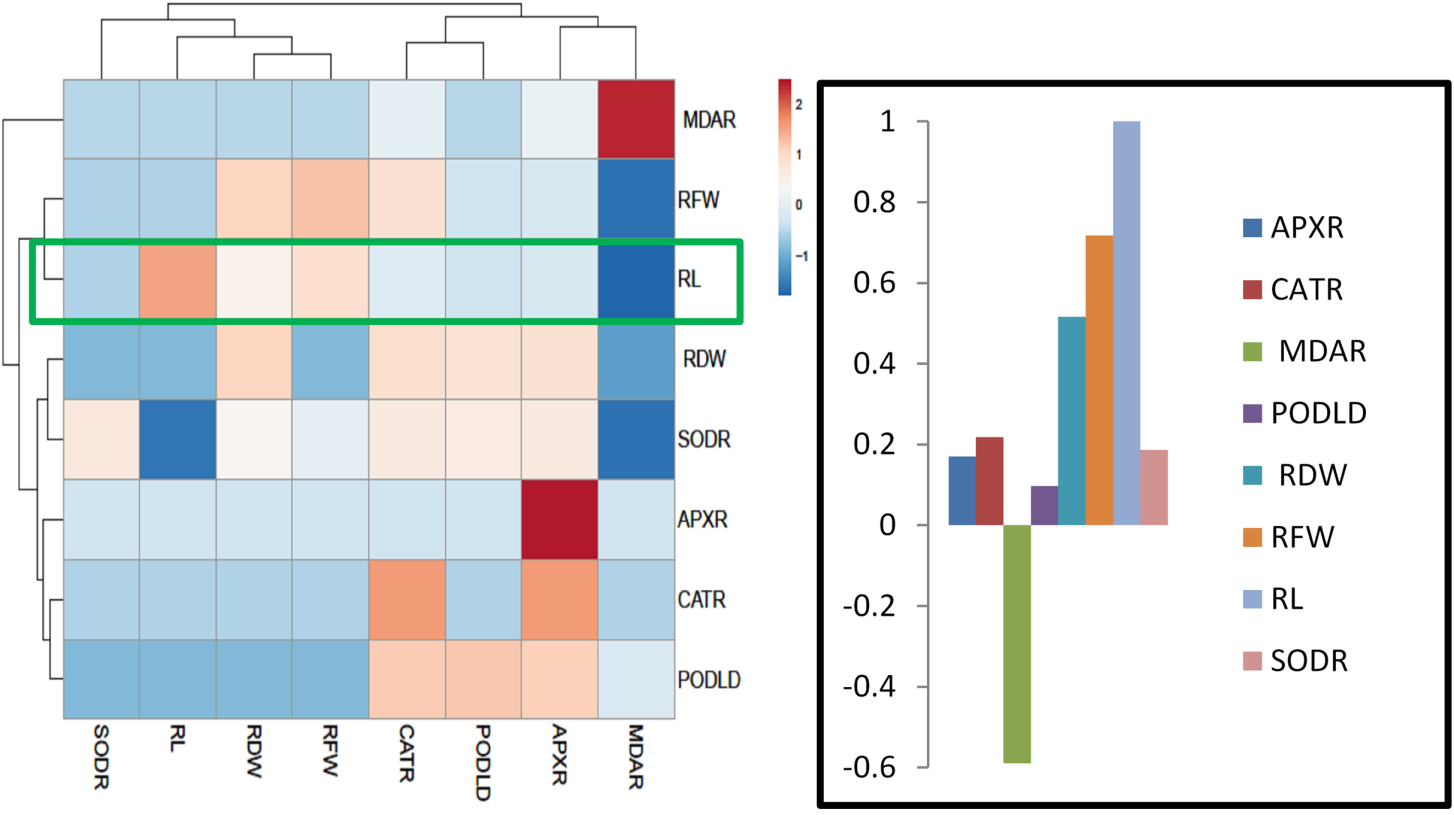
Heatmap responses of Pearson’s Correlation Coefficient (r) for the antioxidant enzymes and growth attributes of wheat roots treated with sole and combined application of SA and BF under drought stressed condition. APXR, Roots ascorbate peroxidase; CATR, roots catalase; PODR, roots peroxidase; SODR, roots superoxide dismutase; MDAR, roots melondialdehyde.

For heat map analysis the data of the wheat seedlings under drought stress were classified as antioxidant enzymes, stress determinants, and RWC and each group showed positive correlations (Fig. 7). A comparison of the factors related to RWC (displayed by green boxes) indicated that RWC of wheat leaves had a positive correlation with photosynthetic pigments and negative correlation with proline and MDA content and SOD, POD, CAT and APX activities. The wheat roots data under drought stress were classified for heat map as antioxidant enzymes and root growth attributes and each group showed positive correlations (Fig. 8). A comparison of the factors related to RL (shown by green boxes) indicated a positive correlation of roots with root fresh weight (RFW), root dry weight (RDW), SOD, POD, CAT, and APX, but a negative correlation with MDA content.

## Discussion

Due to frequent abiotic stresses, especially drought, development and yield of food crops are immensely reduced. Currently, global efforts are underway to develop strategies to increase crop production under drought stress conditions (Soomro et al., 2015). Reduction in growth attributes of wheat plants under drought stress is due to the competition for nutrients and water between roots and shoots (Mehrdad, Sepideh & Hamed, 2017). The main mechanism of plants’ adaptation to drought stress is the inactivation of shoot metabolism to conserve nutrients in roots which assists the root elongation (Danish et al., 2019).

### The mechanism used by bio-fertilizer to alleviate drought stress

The present study is an effort to investigate the effects of SA and BF on drought stressed wheat plants. The bio-fertilizer formulations containing plant-associated microorganisms play an important role in conferring tolerance to abiotic stresses (Yasmin et al., 2019). The results provide evidence that seed coating with BF “phytoguard” consists of a consortium of PGPR strains showing phosphorous and potassium solubilization, nitrogen fixation, siderophore activity and release of PGRs had a significant impact on the growth of wheat under drought stress. The main mechanism through which PGPR/BF alleviates drought stress is the ACC deaminase activity (Danish et al., 2019). Danish et al. (2019) reported that IAA produced by PGPR in the rhizosphere helps plants to increase cell size which results in the production of more ACC by using ACC synthase enzyme. This ACC is excreted by roots in the rhizosphere with other root exudates. ACC deaminase produced by PGPR cleaves this ACC into ammonia and α-ketobutyarate which ultimately inhibit ethylene production and improves root elongation (Danish et al., 2019).

We observed that a significant number of PGPR colonized rhizosphere soil of BF treated wheat plants, under optimal as well as drought. Hassan, Mcinroy & Kloepper (2019) reported that nutrient-rich root exudates secretion from wheat plants including sugars, phenolic compounds; exopolysaccharides, and phyto-siderophore facilitated the bacterial colonization resulting in more water and nutrients uptake. Exopolysaccharide production by PGPR played an important role in maintaining moisture status at the root-soil interface (Sandhya et al., 2009).

Results showed that sole application of BF manifested a 78% increase in protein content under drought stress which becomes 1.8-fold when applied along with SA. These results are in accordance with Lim & Kim (2013) who reported that *B. licheniformis* inoculation in drought exposed pepper plants exhibited more than 1.5 fold induced expression of proteins related to drought stress.

### Imperative role of SA for growth promotion and stress tolerance in plants

Besides the vital role of BF, foliar spray of plant hormone SA also played a positive role in wheat growth promotion. From the results, it is obvious that SA had a stimulatory effect on the plant growth in terms of plant length, fresh and dry weight as well as leaf area. Our findings are consistent with previous reports on the role of SA in defense against pathogen attack, plant growth, and development by modulating physiological and biochemical traits (Klessig, Choi & Dempsey, 2018). Besides, SA activates the biosynthesis of key enzymes that are involved in important metabolic pathways particularly the glyoxylate cycle, the pentose phosphate pathway, glycolysis, and gluconeogenesis (Rajjou et al., 2006).

Salicylic acid has been described to play an important role in plants for resistance and tolerance to abiotic stresses (Khan et al., 2018). The present study confirmed these findings when the foliar spray of SA effectively protected the roots and shoots of wheat from inhibitory effects of drought stress. These findings are directly in line with previous findings where SA plays a key role in plant defense against drought tolerance (Munne-Bosch & Penuelas, 2003; Bandurska & Stroiński, 2005). Miura & Tada (2014) reported that SA dependent PR1 and PR2 genes are induced under drought stress previously known to be only related to responses to pathogens.

### Co-application of SA and BF imparts better drought tolerance in plants

#### Maintenance of relative water content

In the current study, seed coating with BF along with the foliar application of SA gives better results than the sole application of SA or BF for growth promotion and drought tolerance in wheat plants. Our results for biomass and RWC showed that combined treatment of SA and BF performed better under optimal and drought. RWC is considered as the best standard for plant water status. Thus, leaf RWC is a key criterion for the evaluation of tolerance to drought stress in plants (Gonzalez & Gonzalez, 2001). Osmoregulation mostly helps plants in maintaining turgor pressure and preventing water loss which can eventually help plants in better water absorption and enhanced metabolic activities (Schonfeld et al., 1988). Liu et al. (2003) found that exogenously applied SA was involved in reducing water loss by regulating stomatal closure resulting in a higher amount of ROS and Ca^+^ accumulation under drought. Cho et al. (2008) reported that volatile organic compounds (VOC’s) proved to be related to SA signaling, were produced by *Pseudomonas chloroaphis 06* which induced drought tolerance in *Arabidopsis* plant.

#### SA and BF integration help thirsty plants by enhancing photosynthetic pigments

Drought stress severely damages wheat seedlings’ germination and growth. It results in loss of green pigments eventually turning the leaves to yellow and wither. These drought-induced phenotypic changes under drought have a correlation with decreased Chl and carotenoid content as shown in Fig. 3. Reduction in photosynthesis doesn’t only result from stomatal closure but also by denaturation of photosynthetic enzymes by Aasamaa & Sober (2011). However, in this study, both sole and combined treatments of BF and SA showed the enhanced synthesis of these pigments in drought-stressed plants. BF sole increased Chl a by 68% under drought stress. This increase doubled when it was applied in combination with SA. A similar pattern of results was obtained by Khan et al. (2018) who reported that co-application of SA and PGPR exhibited a 3× increase in the photosynthetic pigments of plants under water-scarce conditions. These results are also in line with Noreen, Ashraf & Akram (2012a, 2012b) who found the modulating effect applied by the external application of SA enhanced the photosynthetic capacity and stomatal conductance. Danish et al. (2019) reported significant improvement in photosynthetic pigments and gaseous exchange capacity of wheat plants inoculated with a consortium of PGPR strains under drought. The impact of PGPR to improve photosynthetic pigments has been studied well under drought stress (Garcia et al., 2017; Khan et al., 2018). Others have shown that SA augments the photosynthetic efficiency of plants by regulating the light foraging ability and redox reactions (Rao, Reddy & Reddy, 1997).

#### Combined treatment (SA+BF) augments antagonistic relationship of MDA and proline

Drought stress accelerates the loss of water, deterioration of photosynthetic machinery, and membrane peroxidation (Jaleel et al., 2009). Proline is a widely occurring common compatible solute. It not only helps the plants in osmotic adjustments, enzyme protection, and repair but also as a source of carbon and nitrogen under drought and other stress regimes Ashraf & Foolad (2007). We found that the combined application of SA and BF increased proline contents and resulted in the reduction of MDA under drought-stressed conditions in comparison to non-treated drought-affected plants.

Malondialdehyde is produced due to oxidation of membrane and is considered as an indicator for estimation of the level of membrane damage caused by drought (Yasmin et al., 2019). We found that control plants had lower levels of MDA whereas, drought-affected plants showed an increased amount. Our results are in accordance with the findings of Singh, Jha & Jha (2015), who also reported that reduction in the contents of lipid peroxidase confers drought resistance. Lipid peroxidation of membranes facilitates the stress hormone ethylene to reach the internal organelles like photosynthetic apparatus and inhibit the activities of chlorophyllase enzymes resulting in reduced Chl content and photosynthesis (Khan et al., 2015). Maximum reduction was recorded in the combined application of SA a BF in drought-affected plants in roots and shoots. SA reduced the cellular accumulation of MDA content in stressed plants resulted in reduced membrane damage and ultimately reduce harmful effects to the internal organelles (Fayaz & Bazaid, 2014). *Pseudomonas lini*, *Serratia plymuthica* inoculated plants showed reduced lipid peroxidation in jujube seedlings (Zhang et al., 2020).

#### Combine treatment (BF+SA) induced higher antioxidant enzymes activities

Induction of drought results in enhanced production and concentration of ROS (Hasanuzzaman et al., 2020). These ROS denature proteins, oxidize lipids, and damage deoxyribonucleic acid, resulting in oxidative damage and dysfunction of cells (Hasanuzzaman et al., 2020). In our research, we studied the antioxidant enzyme activities to scavenge the ROS in roots and leaves under drought stress. Plants combat with the ROS through their antioxidative defense system, consisting of both non-enzymatic and enzymatic constituents (Hasanuzzaman et al., 2020).

Taken together, our results demonstrated three key mechanisms of drought tolerance in wheat. First, higher activities of the antioxidant enzymes (SOD, CAT, APX, POD) can help to ameliorate drought stress. These results are in accordance with Zhang et al., 2020 who showed that drought-stressed plants showed a higher amount of antioxidant enzymes in comparison to non-stressed control.

Second, the sole and combined application of SA and BF conferred drought tolerance by promoting the activities of the ROS-scavenging enzymes. Our results are contrary to the findings of Khan et al. (2018) who reported a decreased antioxidant activity when treated with a consortium of PGPR along with SA in chickpea under drought stress. Various studies reported the increase in the activities of ROS in wheat plants inoculated with PGPR (Gusain, Singh & Sharma, 2015, Adesemoye & Egamberdieva, 2013). Furthermore, the role of endogenous and exogenous SA in regulating ROS in a cell is well studied by Kang, Li & Guo (2014) who found an obvious SA dependent regulation of ROS scavenging machinery of plants under abiotic stress. Higher activity of ROS scavenging enzymes (SOD, POD, and CAT) by exogenous application of SA in water-stressed plants was reported by Saruhan, Saglam & Kadioglu (2013).

Third, higher activities of the antioxidant enzymes SOD were observed in roots as compared to the shoots. The analysis provided evidence that shoots and leaves under drought stress depicted more pronounced stress symptoms such as yellowing of leaves and dullness than roots. This may be attributed to the fact that ROS is present in a greater amount in the chloroplast as thylakoid membranes have excited pigments that might interact with O_2_ to form strong oxidants such as O^−2^ (Reddy, Chaitanya & Vivekanandan, 2004).

## Conclusion

The application of BF and SA induced drought tolerance in wheat plants by improving various morphological and physiological traits. Co-application of BF and SA resulted in increased resistance mainly through regulation of ROS scavenging enzymes and thus provided plants with better defense mechanisms against drought stress. This combination can play a significant role in the reclamation of drought-affected lands in arid and semi-arid regions. Further investigations may also help in elucidating the interaction of SA and BF in drought amelioration at the molecular level. Field trials can also validate the practical usability of their combined application in drought tolerance.

## Supplemental Information

10.7717/peerj.9960/supp-1 Supplemental Information 1 Raw data. All the data in triplicates which were statistically analyzed and represented as figures and tables.

## Additional Information and Declarations

### Data Availability

The following information was supplied regarding data availability: Raw data are available as a Supplemental File.
